# Full rescue of an inactive olfactory receptor mutant by elimination of an allosteric ligand-gating site

**DOI:** 10.1038/s41598-018-27790-7

**Published:** 2018-06-25

**Authors:** Kanika Sharma, Sabine Balfanz, Arnd Baumann, Sigrun Korsching

**Affiliations:** 1 Institute of Genetics, Biocenter, University at Cologne, Zülpicherstrasse 47a, 50674 Cologne, Germany; 2 Institute of Complex Systems (ICS-4), Research Center Jülich, 52428 Jülich, Germany

## Abstract

Ligand-gating has recently been proposed as a novel mechanism to regulate olfactory receptor sensitivity. TAAR13c, the zebrafish olfactory receptor activated by the death-associated odor cadaverine, appears to possess an allosteric binding site for cadaverine, which was assumed to block progress of the ligand towards the internal orthosteric binding-and-activation site. Here we have challenged the suggested gating mechanism by modeling the entry tunnel for the ligand as well as the ligand path inside the receptor. We report an entry tunnel, whose opening is blocked by occupation of the external binding site by cadaverine, confirming the hypothesized gating mechanism. A multistep docking algorithm suggested a plausible path for cadaverine from the allosteric to the orthosteric binding-and-activation site. Furthermore we have combined a gain-of-function gating site mutation and a loss-of-function internal binding site mutation in one recombinant receptor. This receptor had almost wildtype ligand affinities, consistent with modeling results that showed localized effects for each mutation. A novel mutation of the suggested gating site resulted in increased receptor ligand affinity. In summary both the experimental and the modeling results provide further evidence for the proposed gating mechanism, which surprisingly exhibits pronounced similarity to processes described for some metabotropic neurotransmitter receptors.

## Introduction

**Figure 1:**
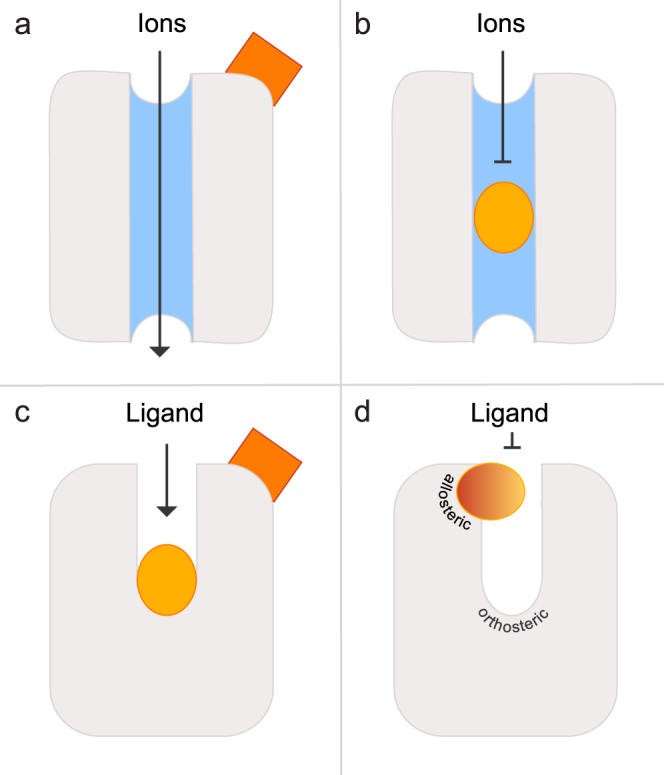
Schematic illustration of allosteric and orthosteric sites in ligand-gated ion channels and GPCRs. (**a**) Binding of an allosteric ligand (reddish) to the extracellular binding site of a ligand-gated ion channel induces a conformational change causing opening of the ion-conducting pore. (**b**) Binding of an antagonist (dark yellow) in the pore blocks the channel and can lead to its deactivation. (**c**) An orthosteric binding site is located inside a cavity formed by transmembrane domains in class A GPCRs. Binding of a ligand (reddish) to an allosteric site topographically distinct from the orthosteric site eventually can modulate binding of the orthosteric ligand (dark yellow). (**d**) Allosteric binding sites in some GPCRs are located externally and almost above the orthosteric binding site. Binding of a ligand (bicolor) to this site could block ligand access to the internal binding-and-activation site.

Gating of ion channels constitutes a central feature of ionotropic neurotransmitter receptors, also dubbed ligand-gated ion channels (LGIC)^1–3^. Ligands like acetylcholine, glutamate, GABA or glycine usually bind far away from the channel domain. Upon binding ligand-induced conformational changes result in opening of the channel pore (Fig. 1a)^4–6^ and allow ions to traverse the channel. Allosteric effects on receptor activation are well known for metabotropic receptors as well: receptor-ligand interaction far away from the binding-and-activation site (the orthosteric site) can control receptor activity, e.g. by locking the receptor in an inactive conformation^7^.

However, an alternative mechanism exists, in which an LGIC ligand binds inside the channel pore, thus blocking the channel (Fig. 1b). Metabotropic receptors (GTP-binding-protein coupled receptors, GPCRs) lack a channel pore, but exhibit a related feature^8^. The ligand binding-and-activation site of class A GPCRs^9^ is usually located in a cavity formed by the transmembrane domains of the receptor (Fig. 1c). Therefore a ligand, starting at the extracellular face of the receptor, has to traverse an entry tunnel to gain access to its final binding-and-activation site. This geometry potentially enables ligands to block the entry tunnel thereby regulating GPCR signaling (Fig. 1d). Although a few publications have addressed this possibility, an allosteric modulatory site located above the orthosteric binding-and-activation site has only been described for muscarinic acetylcholine receptors (mAChR)^10,11^ the occupied state, this site might be properly positioned to obstruct the ligand entry tunnel.

Usually, allosteric and orthosteric binding sites are occupied by different ligands. For mAChRs, however, a weak binding of acetylcholine and other agonists to an allosteric binding site has been deduced from complex binding studies^12^. In this sense an allosteric ligand binding site positioned above the orthosteric binding-and-activation site would be well suited to regulate ligand access to the latter.

While a recent molecular dynamics simulation of the β2 adrenergic receptor (β2AR) has not addressed the case of ligand-gating, a transient arrest of alprenolol has been observed at a weak binding site in an outer ‘vestibule’ of the protein, before the ligand traverses to its final binding-and-activation site^13^. Pausing at a weak binding site will result in a slower ligand on-rate at the binding-and-activation site and eventually result in a lower apparent affinity. An allosteric ligand binding site thus opens a fascinating possibility for receptor evolution to fine-tune the affinity of GPCRs according to the respective functional requirements without affecting the intricate binding-and-activation machinery.

The vast majority of GPCRs are members of the chemosensory receptor family (olfactory and taste receptors)^14^, and the large majority of these are assigned to the class A clade of GPCRs^9^. Recently the possible existence of such allosteric ligand-gating mechanisms in chemosensation has been considered for a member of the trace amine associated receptor (TAAR) family^15–18^ of olfactory receptors from zebrafish, TAAR13c. The binding-and-activation site was shown its position to be located about one third into the cavity formed by the transmembrane domains, an expected position for a ligand binding-and-activation site in class A GPCRs. Additionally, a weaker binding site in an outer vestibule was described, which appeared to be properly located to block access of cadaverine, the native ligand of TAAR13c, to the internal binding-and-activation site^19^. Destruction of this outer binding site resulted in a supersensitive mutant^19^ leading us to suggest that this site controls ligand entry to the binding-and-activating site of the receptor. This is a novel mechanism for chemosensory receptors, and to the best of our knowledge has not been examined in these terms in any GPCR. Thus we sought independent proof and stringently tested our hypothesis both with additional mutations and with more specialized modeling approaches.

We report here that a novel mutation of the potential gating site (D279R) shows significantly enhanced affinity compared to the wildtype receptor, consistent with the suggested gating function. In an attempt to understand the potential interaction between the binding-and-activation site and the gating site we generated a double mutant (D112E/D279N), which fully rescued the severe loss in binding ability of the binding-and-activation site mutant (D112E) to wildtype levels, consistent with the individual mutations sequentially affecting the overall affinity of the receptor. Furthermore, two independent modeling approaches confirmed the existence of the predicted allosteric ligand-gating site in a vestibule at the extracellular face of TAAR13c. Our findings suggest that a ligand-binding external vestibule may be a much more widespread regulatory feature of class A GPCRs than previously assumed. If so, future attempts at structure-based rational drug design in class A GPCRs may be well advised to focus on modifications of this external niche. Even where such an external vestibule is not present, modeling-guided mutations may be used to introduce novel allosteric binding sites bordering the entry tunnel for receptor ligands.

## Results

Previous studies have identified zebrafish TAAR13c as the highly sensitive and specific receptor for cadaverine^17^. The docking of cadaverine to the binding-and-activation site of TAAR13c was shown to engage two aspartates, D112^3.32^ in transmembrane region (TM) 3 and D202^5.42^ in TM5 which form salt bridges with the two positively charged amino groups of cadaverine^19,20^. Some mutations of TAAR13c had unexpectedly resulted in the generation of supersensitive mutants, and the loss of a gating site (D279N) impeding ligand entry to the receptor’s binding-and-activating site has been hypothesized as a possible explanation^19^. The proposed gating site appeared to serve as a ligand-binding site on its own and in its occupied form seemed to block ligand entry. Since this amounts to a highly unusual mechanism for chemosensory receptors, we challenged the hypothesis here in several ways. Firstly, we employed two advanced modeling approaches to identify the tunnel allowing ligand passage to the binding-and-activation site of TAAR13c. These algorithms were more robust and versatile than general-purpose molecular visualization programs used previously to assess binding pockets of receptor proteins. Secondly, to gain independent support for our hypothesis we introduced additional mutations into TAAR13c. The mutant receptors were constitutively expressed in cell lines and examined pharmacologically. Furthermore, ligand-interaction of these receptor mutants was modelled and compared to the wildtype receptor.

### The predicted ligand-gating site lines the entry tunnel for cadaverine in TAAR13c

**Figure 2:**
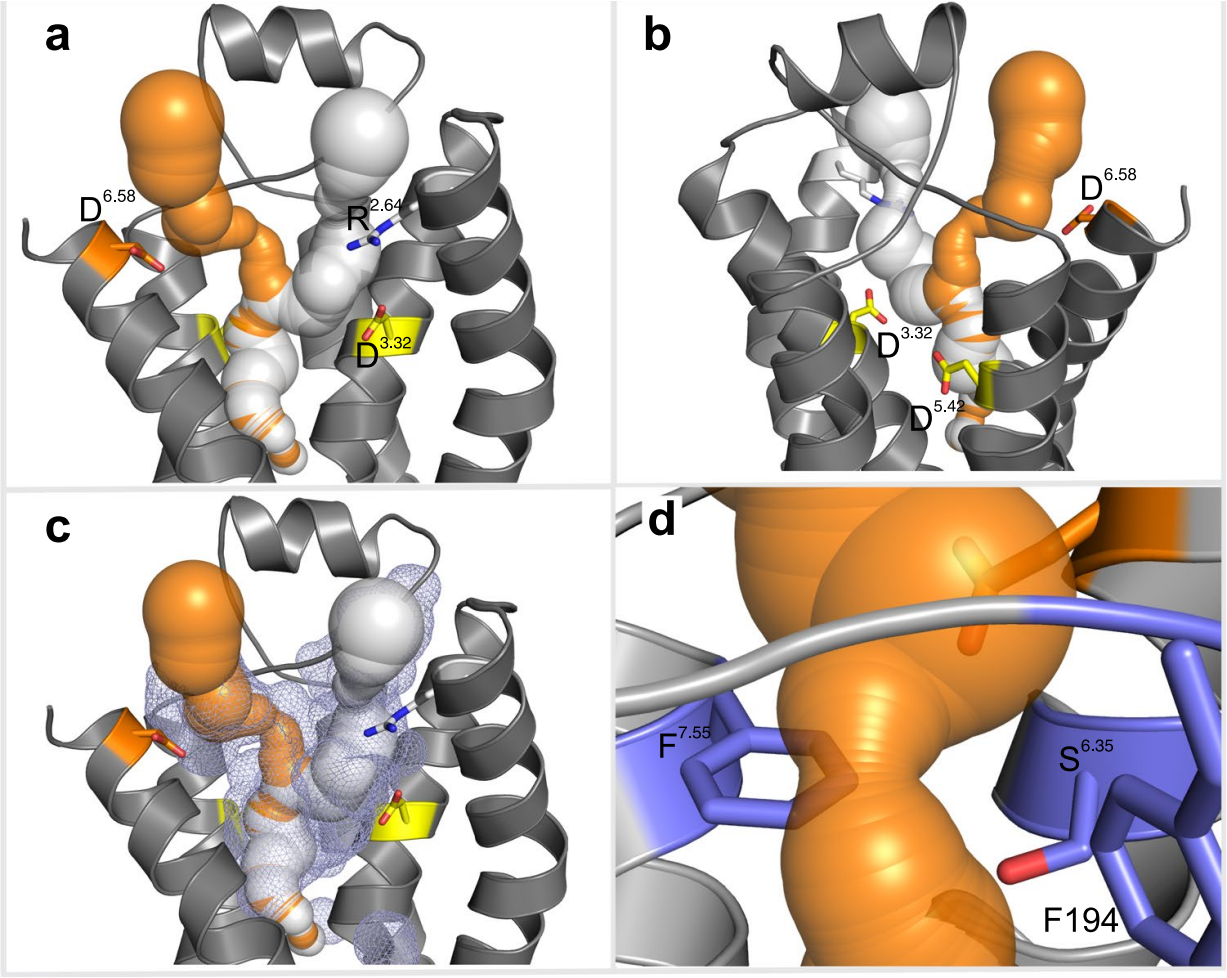
Prediction of ligand access tunnels in TAAR13c by MOLE2.0 shows presence of a bottleneck. (**a**) Ligand access tunnels predicted by MOLE2.0 were imposed on a cartoon representation of the TAAR13c structure, leading towards the internal ligand binding-and-activation site. The negatively charged aspartic acid residue D279^6.58^ in TM6 lines a tunnel (orange) through which cadaverine passes on its way to the internal binding site. The positively charged arginine residue R92^2.64^ in TM2 impedes ligand entry via an alternative tunnel (white). (**b**) View of panel (**a**) from the back; residues D112^3.32^ and D202^5.42^ (yellow) in TM3 and TM5 are part of the ligand binding site. (**c**) Both ligand access tunnels are located inside the central cavity (blue mesh). (**d**) Zoom-in on the bottleneck of the tunnel shown in (**b**) with a radius of 1.53 Å. Residues F194, F291^7.55^, and S276^6.35^ (blue) are shown facing the bottleneck.

Previously we had predicted^19^ the existence of a ligand-gating site near the extracellular surface of the TAAR13c receptor, using PyMol^21^, a general purpose molecular visualization tool. Aiming to independently test this prediction, here we employed MOLE2.0^22^, a more advanced and robust approach compared to PyMol. With MOLE2.0 the cavities and potential tunnels allowing the ligand to enter the receptor, contact the gating site, and traverse towards its final binding site were identified. Two tunnels were predicted in the upper third of TAAR13c TM domains (Fig. 2a). Both are buried inside the protein cavity (Fig. 2c) and lead towards the internal ligand binding-and-activation site (Fig. 2b). One of the tunnels in TAAR13c is 26.0 Å long and situated between TM 2, 3, and 7. At its entrance, it is lined by a positively charged arginine residue, R92^2.64^ (Fig. 2a). The presence of this positive charge might disfavor this path for the positively charged cadaverine to enter the receptor and to reach the ligand binding-and-activation site. The second tunnel is shorter (20.6 Å) and located between TM 3, 5, 6 and 7. It is lined by the previously identified ligand gating residue, D279^6.58^ at its entrance (Fig. 2a). This negative charge around the entrance probably serves as an attractant for cadaverine, thus making this path more favorable to reach the ligand binding-and-activation site.

### Cadaverine has to traverse a bottleneck after passing the gating site

The tunnel predicted by MOLE2.0 for the cadaverine path features a pronounced constriction (Fig. 2d), which is mainly formed by three residues: F194 in ECL2, F291^7.55^, and S276^6.35^. The arrangement of these three residues creates a ‘bottleneck’ of 3.06 Å in diameter, which is by far the narrowest part along the entire path. Similar bottlenecks have also been observed in opsin as well as in several ion channels^23,24^.

Docking simulations (described below) for cadaverine never showed a binding interaction for the constriction site. Thus, cadaverine moves from the extracellular region to the internal binding-and-activation site without pausing at the constriction. Since cadaverine is a straight chain diamine with a cross section diameter of 3 Å, its molecular properties should allow it to squeeze through the bottleneck, especially when considering the rotatable single carbon-bonds in the backbone. We also observed the constriction in the TAAR13c mutants described below and those studied previously (Table 1). Interestingly, in all mutants, including the super-sensitive ones^19^, the constriction is even more pronounced than in the wildtype receptor (Table 1), further consistent with no deleterious influence on the affinity of TAAR13c.

**Table 1:** Bottleneck diameter and contact distances for cadaverine amino groups with D112^3.32^ and D202^5.42^ for mutant and wildtype TAAR13c predicted by TomoDock.

Wt/Mutant	Bottleneck diameter in Å	TomoDock distance of Cad-NH_2_ with	
		112	202
TAAR13c	3.06	3.1	3.0
D279R	2.06	3.2	3.0
D112E/D279N	2.16	9.0	3.1
D279N	2.14	3.0	3.3
D112E	2.12	9.0	3.1
D279E	2.12	—	
D279A	2.14	—	

### Multistep docking algorithm suggests cadaverine lingering at the gating site before its progress to the internal binding-and-gating site

**Figure 3:**
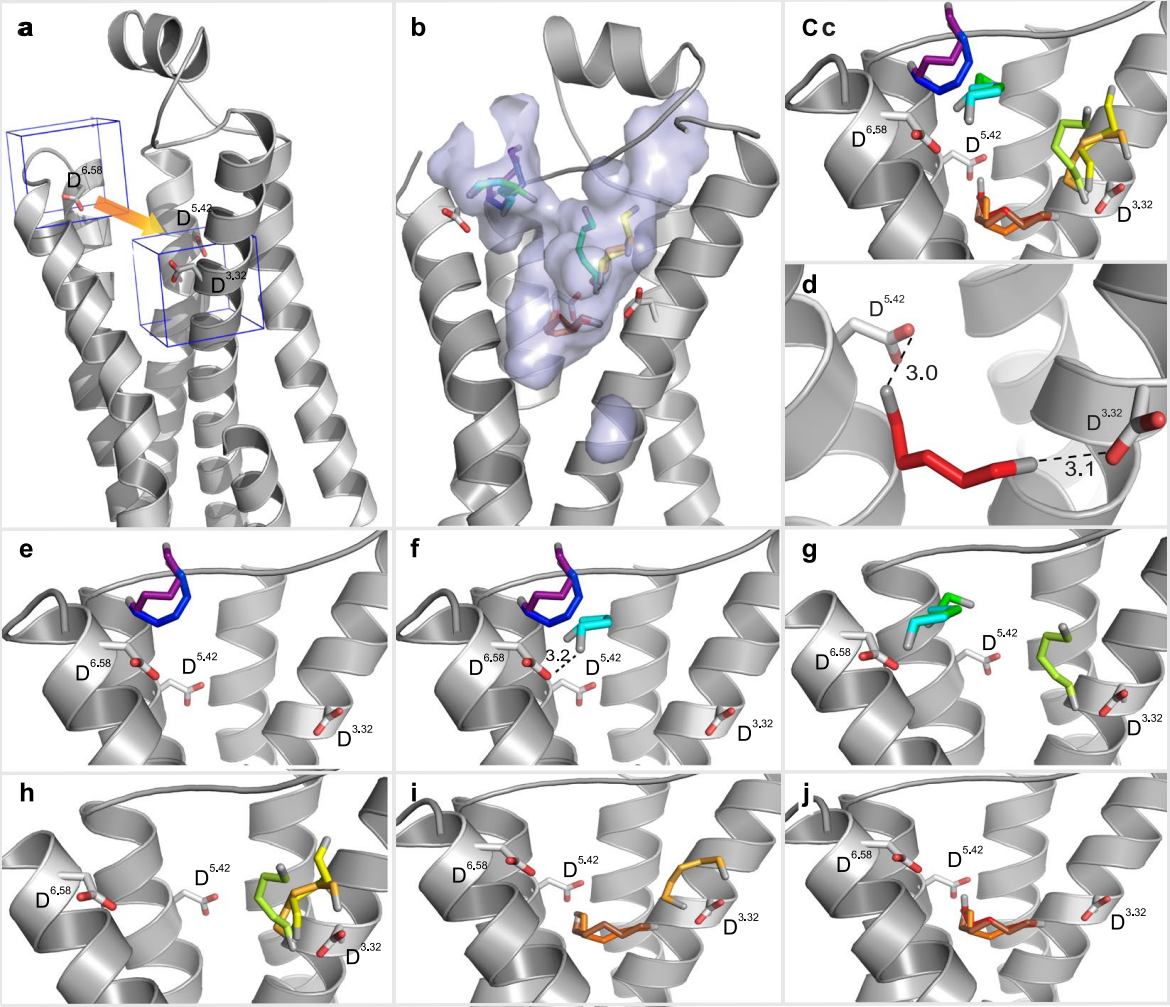
Binding path of cadaverine predicted by TomoDock exhibiting different cadaverine poses. (**a**) A cube (in blue line) encompassing gating site residue D279^6.58^ in TM 6 shows the first search space for Tomodock. The last search space is visualized by the second cube containing D112^3.32^ and D202^5.42^. Intervening search spaces are intercalated at 1 Å steps along the orange arrow. (**b**) Surface view of the largest cavity in TAAR13c. Predicted cadaverine binding positions inside this cavity range from the extracellular protein surface to the internal ligand binding-and-activation site and are shown as sticks in rainbow colors (magenta to red). (**c**) Zoom-in on panel (b) without displaying the cavity to enable a clearer view on the intermediate binding positions of cadaverine along its path. (**d**) Final binding position of cadaverine with the main interacting residues (D112^3.32^; D202^5.42^) and the distances in Å from the carboxyl groups of these residues to the amino groups of cadaverine. Salt bridges are visualized as black dashed lines. (**e**) Progressive docking showing the first two steps of different orientations of cadaverine entering the cavity. Progress in ligand entry is shown in rainbow colors. (**f**) Step 3 of cadaverine (cyan) interacting with D279^6.58^ in TM6. (**g**) Step 4 of the cadaverine (dark green) path. In step 5 (light green) cadaverine leaves its previous position and slides to approach aspartate D112^3.32^ of the binding site. Step 1 and 2 are hidden for clarity. (**h**) Steps 6 and 7 (yellow and orange) show cadaverine exploring the D112^3.32^ area without any major interactions. Step 1–4 are hidden for clarity. (**i**) In step 8 and 9 (orange and brown) cadaverine is approaching the binding site. Cadaverine turns in order to establish binding to D112^3.32^ and D202^5.42^. Steps 1–7 are hidden for clarity. (**j**) Final step of docking (red) showing cadaverine inside the binding site with its main interacting residues, aspartate D112^3.32^ and D202^5.42^.

In order to study interactions of cadaverine as it moves along its path inside the receptor, we used the TomoDock algorithm^25^. The path towards the binding-and-activation site of TAAR13c is arbitrarily divided into a number of segments. Each segment is treated individually for the docking step thereby allowing the detection of local optima for ligand position and interaction with the receptor. For each segment we defined a cubic search space that moves from the top of the tunnel to the bottom in steps of 1 Å length. The starting point of the docking simulation is the very beginning of the tunnel flanked by the gating site (D279^6.58^) at its bottom and then the position of segments gradually progresses towards the internal ligand binding-and-activation site of the receptor (Fig. 3a). The final step encompasses the key interacting residues of the internal binding-and-activation site, D112^3.32^ and D202^5.42^ (Fig. 3a). TomoDock assigns a low numerical score to ligand orientations which allow an interaction with the receptor. Consequently, for each step along the migration path of cadaverine we chose the cadaverine orientation with the lowest score.

At the first step of docking, cadaverine is located at the rim of the tunnel entry surface, which constitutes an “external niche”. Inside this niche, cadaverine engages with S199^5.39^ in TM5 and Y193 to form a hydrogen bond, as well as F194 and E175 in ECL2 to stabilize the backbone (Supplementary Table 2). In the second step, as cadaverine moves further inside from the cavity mouth (Fig. 3e), the contact with E175 is lost while the others are retained. In step 3, cadaverine moves towards the gating site residue, D279^6.58^, which resides near the external end of TM6, resulting in an interaction between the carboxyl group of D279^6.58^ and an amino group of cadaverine (Fig. 3f). This results in the occupation of the hypothesized gating site. In the next step, there isn’t much change in the position of cadaverine (Fig. 3g). Thus, cadaverine appears to pause at the gating site in the external niche, before it slips through the bottleneck between ECL2, ECL3 and TM5–7 (Fig. 3g). In the next step (step 5) cadaverine has passed the constriction, approaching D112^3.32^ in TM3 (one of the two aspartate ‘anchors’ of the internal binding-and-activation site) to form a salt bridge (Fig. 3h). In steps 6 to 7, cadaverine stays around D112^3.32^ by adopting various orientations (Fig. 3h). A common interaction in steps 5–7 is a salt bridge with D112^3.32^ and a polar interaction with neighboring residue Y299^7.43^ in TM7. Deep inside the binding site, cadaverine changes its orientation slightly in step 8 and 9 (Fig. 3i,j). Finally in step 10, for the first time cadaverine makes a contact with D202^5.42^ in TM5 to attain the most favorable binding position with two salt bridges (Fig. 3j). One amino group of cadaverine forms a salt bridge with D112^3.32^ with a distance of 3.1 Å. The other amino group forms a salt bridge with D202^5.42^ with a distance of 3.0 Å (Fig. 3d) in the final docked position. The results obtained from this docking simulation are in close agreement with our previous data^19^ (Table 1). Taken together, the stepwise docking algorithm employed here provides a plausible path for cadaverine to traverse the receptor from the gating site to the internal binding-and-activation site. Furthermore, the close similarity to earlier predicting confirms the shape and location of both external gating and internal binding-and-activation site.

### Abolishing the gating site by introducing a ligand-repellent charge results in significantly increased receptor affinity

**Figure 4:**
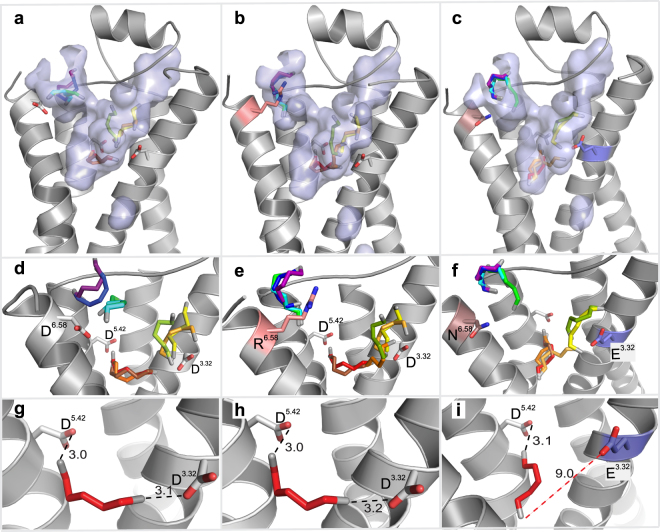
Binding path analyses of TAAR13c mutants indicate effects limited to the gating-site. Panels (a,d and g) show the wildtype and are taken from Fig. 3(b–d). (**b**) Surface view of the largest cavity in the D279R mutant. The mutated residue, R279^6.58^ is shown in pink. Successive progress in ligand entry is shown in rainbow colors. (**c**) Surface view of the largest cavity in the double mutant D112E/D279N. Mutated residues, E112^3.32^ and N279^6.58^ are shown in brown and blue, respectively. Progress in ligand entry is shown in rainbow colors. Panels d–f are zoom in on panels a–c without cavity showing the various orientations of cadaverine at different time points in rainbow color. (**e**) The introduced arginine residue R279 is shown in salmon. (**f**) The introduced asparagine and glutamate residues are shown in brown and blue respectively. Panels g–i show final binding position of cadaverine inside binding site with final binding position of cadaverine. The distances between the carboxyl groups of the aspartate(s) and the amino group(s) of cadaverine are given in Å. Salt bridges are visualized as black dashed lines. (**h**) Wildtype-like docking of D279R mutant. (**i**) Cadaverine in the binding site of the D112E/D279N mutant. The distance of E112^3.32^ from cadaverine is 9.5 Å (red dashed line) and thus too large to participate in ligand binding.

**Figure 5:**
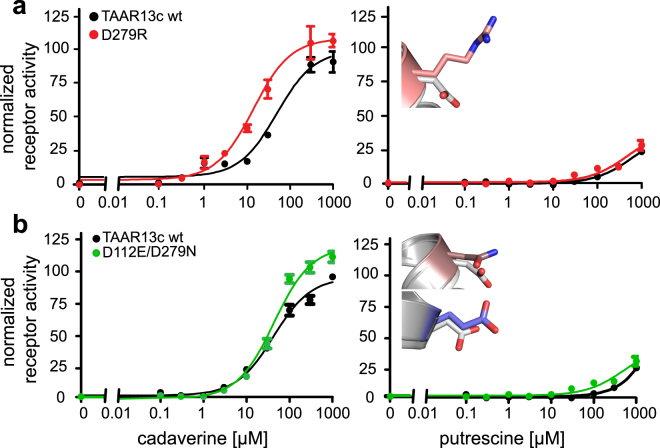
Concentration-response curves of TAAR13c mutants. HEK293T cells were stably transfected with TAAR13c constructs either altered by single or double point mutations. A cell line expressing TAAR13c wildtype receptors was included for control. Cadaverine and putrescine were added in the range of 0.1 µM to 1 mM. The resulting calcium increase was detected by Fluo-4 and calculated as ∆F/F and normalized to the value obtained with 10 µM NKH477, an agonist of membrane-bound adenylyl cyclases. Left column, response to cadaverine; right column, response to putrescine. Representative binding curves shown for the wildtype receptor and mutants (TAAR13c wildtype, black; D279R, red; D112E/D279N, green). Right panel, insets show mutant side chains (D279N, pale brown; D279R, pink; D112E, blue) overlaying the wildtype residue D in grey. (**a**) The cadaverine affinity of the D279R mutant was approximately 5fold higher compared to wildtype. (**b**) The double mutant D112E/D279N had a similar cadaverine affinity as the wildtype receptor with a slight increase in efficacy. For putrescine the affinities of mutant and wildtype TAAR13c receptors were also similar.

In order to further explore the function of the gating site residue, we substituted the negatively charged D279^6.58^ in TM6 for a positively charged arginine residue, creating a D279R mutant (Fig. 4b). The major differences in this mutant are the loss of the salt bridge with D279, i.e. the destruction of the gating site, which should facilitate the entry of cadaverine into TAAR13c, and, on the other hand, the introduction of a ligand-repellent charge, which should hinder the approach of cadaverine towards the external niche. Experimentally, we observed that the apparent affinity of the mutant is 5.2 times higher than for the wildtype receptor (Fig. 5a and Supplementary Table 1), but significantly below the previously reported supersensitive mutant D279E^19^. This result is consistent with the assumption that the loss of the gating site overrides ligand repulsion by the positively charged arginine residue in the D279R mutant. Similar results were observed for mutant TAAR13c receptors, when putrescine was applied as a ligand (Fig. 5a).

The D279^6.58^ residue is located on TM6 and close to ECL3. In many GPCRs this site appears to control receptor activity via ligand interactions^26–30^. Future analyses will have to show, whether the influence of residue 6.58 is conserved in other olfactory receptors.

### Path simulation and multistep docking show a localized effect of the gating site mutation

Next, we examined, whether the experimentally observed increase in ligand affinity of the D279R mutant could be corroborated by modeling the D279R mutant with a multistep docking procedure, using TomoDock, as described for the TAAR13c wildtype receptor. We found that in steps 1–4, cadaverine is located in the external niche close to the cavity opening (Fig. 4e). Interactions were found between cadaverine’s amino groups and polar amino acid residues, Y193, S199^5.39^, and E175 as well as the backbone carbonyl oxygen atom of F194 (Supplementary Table 2). These interactions are very similar to those observed in the wildtype receptor (see above). In other words, the external niche seems to have a very similar morphology and even retains the minor binding interactions in the mutant. Thus, the observed super sensitivity seems to originate primarily from the isolated loss of the salt bridge interaction and the ensuing reduction in binding affinity.

In step 5, cadaverine starts entering the internal binding pocket by squeezing through the constriction, which is in fact narrower than in the wildtype receptor (Table 1). Before reaching the final docked position, within the internal binding-and-activation site, cadaverine adopted several alternative orientations (Fig. 4e), all of which possessed the salt bridge between the cadaverine amino group and the carboxyl group of D112^3.32^, before relaxing into the final docked pose. Upon reaching the internal binding-and-activation site in step 10, cadaverine orients itself to form stable salt bridges with D112^3.32^ and D202^5.42^ at a distance of 3.0 Å and 3.2 Å respectively (Fig. 4h). The distances are very similar to those calculated for wildtype TAAR13c (Table 1). The results suggest that the loss of the ligand gating site may have a conformational effect on the bottleneck but no or rather minute influence on the internal ligand binding-and-activation site. Constriction sites in ligand entry tunnels are not uncommon and seem to have surprisingly little influence on ease of a ligand’s passage to its final binding site^13,31^.

Taken together, we conclude that even the rather drastic exchange of a negative for a positive charge at the gating site has mostly local effects, which can be understood in terms of a balance between the loss of the external ligand-binding site and the gain of a repulsive force at the tunnel entrance.

### Full rescue of an inactive TAAR13c mutant by elimination of the gating-site

The internal binding site for cadaverine is formed by two aspartate residues, D112^3.32^ and D202^5.42^ as the main interacting partners. In our previous docking and mutagenesis study we confirmed that both residues are essential for cadaverine binding and activation of TAAR13c. Removal of either residue resulted in total loss of observable receptor activity^19^. On the other hand, elimination of the external gating-site resulted in supersensitive receptors^19^.

In order to understand the balance between the external gating and internal binding-and-activating site, we simultaneously mutated the gating site and one residue of the internal binding site (D112E/D279N; Fig. 4c). In contrast to the inactive D112E mutant^19^, we observed that the double mutant (D112E/D279N) displayed a cadaverine affinity slightly less but not significantly different from the wildtype receptor (Fig. 5b and Supplementary Table 1). Similarly, putrescine activated the double mutant and the wildtype receptor comparably well (Fig. 5b), whereas no activation of the D112E mutant was detected with this ligand^19^.

Inspecting the docking path for the D112E/D279N mutant, cadaverine follows the same spatial route like in the wildtype TAAR13c or the D279R mutant receptors. It pauses at the external niche interacting with F194, Y193, S199^5.39^, and E175 (Supplementary Table 2). After traversing the constriction, which is of similar size as in all other mutants (Table 1), cadaverine stays briefly close to E112^3.32^ and forms transient interactions (Fig. 4f) before it finally reaches the mutated internal binding-and-activation site (E112^3.32^/D202^5.42^). The carboxyl group of E112^3.32^ is displaced by 9.5 Å relative to the amino group of cadaverine, which is too far away to form a salt bridge (Fig. 4i). Thus, in the final orientation cadaverine can only form a salt bridge with D202^5.42^ at a distance of 3.2 Å but the carbon backbone of cadaverine is stabilized by (as in wildtype) L113^3.33^, T116^3.36^, T203^5.43^, F272^6.51^, and W269^6.48^ all situated within 5 Å distance and hence within the range of van der Waals interactions (Supplementary Table 2). Thus in the mutant, the binding pocket retains most of the minor binding interactions and one of the two salt bridges. Surprisingly, this (compared to wildtype) seriously weakened binding in combination with facilitated ligand access due to gating site loss seem sufficient to activate the receptor at similar efficacy as wildtype TAAR13c. Taken together, the results suggest that the outcome of concomitant mutations of gating and internal binding sites can be understood as resulting from linear additivity of localized effects.

## Discussion

Elucidating the molecular mechanism of ligand-to-receptor binding is of central importance to understand cellular signaling, not least because of its large relevance for biomedical research. However, due to the intrinsically dynamic nature of ligand-receptor interactions, little is known about the precise processes by which ligands bind to their receptors, even for GPCRs, which are among the most intensively studied groups of membrane proteins. In class A GPCRs the ligand binding-and-activation site is usually situated inside the transmembrane domain of the receptor, and the ligand has to traverse an entry tunnel to gain access to its destined location^8^. Such a geometry may also enable counteracting mechanisms similar to that of channel blockers in LGICs, where an external (allosteric) binding site for a ligand might block access of ligands to the final binding-and-activation site (orthosteric site) by physically blocking the entrance tunnel (Fig. 1). Evidence for such a mechanism indeed has been obtained for several neurotransmitter and hormone receptors of the class A GPCR group even though it has not been “discussed” in those terms^11,32^. We have recently proposed a ligand-gating mechanism for an olfactory receptor-ligand pair, i.e. TAAR13c-cadaverine^19^. Since this phenomenon had not been described for any chemosensory receptor previously, we have tested our hypothesis with new mutations and modeling algorithms.

We used MOLE2.0^22^ to predict a tunnel paths accessed by cadaverine from the extracellular surface of TAAR13c to the internal (orthosteric) binding-and-activation site. Multistep docking was employed to visualize transient positions of cadaverine, as it moves along the tunnel. Further, to gain independent support for our hypothesis we introduced two additional mutations into TAAR13c, constitutively expressed the proteins in cell lines and investigated their effect(s) on receptor activation by cadaverine.

### A generalized ligand-gating function for position 6.58 at the external end of TM6

Both in mutation analysis and in modeling an aspartate residue at the external end of TM6, position 6.58 (D279 in TAAR13c), emerged as key residue of an external niche serving as the initial binding site for cadaverine. Loss of D279^6.58^ and consequentially loss of the salt bridge to the amino group of cadaverine resulted in a supersensitive receptor, even when, as in the present case, the opposite charge was introduced (D279R) mutation. The increase in affinity of the D279R mutation is severalfold smaller than that previously observed for the D279E and D279N mutations, which is plausible if position 6.58 is ‘gating’ the access of the ligand to the internal binding-and-activation site. Thus, both quality and quantity of the affinity change provoked by the D279R mutation support the gating function of D279^6.58^ and its surrounding niche. As long as a cadaverine molecule is bound to D279^6.58^, the passage from the extracellular surface towards the internal binding-and-activation site is blocked.

Exactly the same position, 6.58, appears to fulfil a similar function in several neurotransmitter and hormone receptors^30^, beta adrenergic receptors^13^ and the gonadotropin releasing hormone receptor^27,33^. In a recent modelling study on two human TAARs, 6.58 was hypothesized to serve as floodgate to remove the solvent shell from the ligand, before it reaches the internal binding site^34^.

### Only one of two entry tunnels is used in TAAR13c

Through which pathway does a ligand enter and exit the internal (orthosteric) binding-and-activation site of TAAR13c? Our results suggest that TAAR13c has two putative ligand entry tunnels. The outer opening of one of these tunnels harbors the previously identified external binding site, D279^6.58^ ^19^. D279^6.58^ along with neighboring polar and/or charged amino acids, confers an electronegative environment to the tunnel which is favorable to attract positively charged cadaverine (Supplementary Fig. 2). The opening of the second tunnel is bordered by a positively charged arginine residue (R92^2.64^), which is less favorable for cadaverines’ entry. However in D279R mutation of the first tunnel introduces a very similar charge environment and still results in a considerable increase in affinity suggesting in reverse that the second tunnel is not able to provide significant ligand access due to additional restraints beyond its external positive charge.

To explain the experimentally observed super sensitivity of the D279R mutation several aspects need to be taken into account. Foremost, there is a destruction of the gating-site. Losing the ability of forming a salt bridge in the external niche should strongly reduce immobilization of cadaverine at this site and thus facilitate its entry into the tunnel. Secondly, cadaverine approaching the D279R mutated receptor could be hindered in entering the tunnel opening due to repulsive electrostatic forces. However, once cadaverine has passed the arginine residue it could even be pushed into the entry tunnel by the same electrostatic force. Additionally, the bulky side chain of arginine narrows the cavity of the external niche compared to the wildtype receptor (Supplementary Fig. 1). This could limit the exploration time for cadaverine, again speeding up its entry into the tunnel. Although these are plausible explanations, the current data unfortunately do not allow to unequivocally define the relative contribution of the individual processes.

Interestingly, beta adrenergic receptors, which share close structural similarity to olfactory receptors also possess two tunnels providing ligand access to the internal binding site^35^. Both tunnels are lined by polar residues and thus are equipped to attract positively charged ligands. In ligand free conformation, however, only one of these tunnels seems to support ligand entry^36^ similar to our findings reported here.

### A constriction in the entry tunnel is neither rate-limiting in TAAR13c nor in other GPCRs

After entering the external niche and prior to proceeding towards the internal binding-and-activation site, cadaverine has to pass a narrow constriction in the tunnel. When comparing the diameter of this constriction in TAAR13c wildtype to current and previously described mutants^19^, we found no obvious correlation with the experimentally determined changes in receptor affinities. In particular, the supersensitive mutants D279N/E/A possess a narrower constriction than the wildtype receptor (see Table 1). These observations seem to indicate that the rate limiting step for cadaverine to reach the internal binding-and activation site is not or only mildly affected by the dimension of the constriction.

A constriction like the one in TAAR13c has been described for some other class A GPCRs, and, like in our case, has not been found rate-limiting for ligand access. For opsin a ‘bottleneck’ structure of similar diameter (3.2 Å) has been described, but the rate at which retinal reaches the binding site is independent of the ease of passage through this constriction^31^. Also for beta adrenergic receptors it was suggested that ligands, before entering the binding pocket had to squeeze through a narrow passage but, again, this was not the rate-limiting step for receptor activation^13^.

### Cadaverine pausing in the external niche may be a representative of a general mechanism regulating ligand access in GPCRs

In this study we have challenged our hypothesis of a ligand-gating mechanism in an olfactory receptor by generating novel mutations as well as calculating the ligand path in relation to the proposed gating-site using more refined modeling approaches. The experimental and theoretical results were consistent and both provided evidence for gating by an allosteric binding site lining the entry tunnel. The multistep docking approach enabled us to visualize the path taken by cadaverine to travel from the extracellular niche to the internal binding-and-activation site. During this movement cadaverine adopts various orientations in the extracellular niche before it proceeds towards the internal binding site in both wildtype and mutant receptors. Very similar results have been obtained for beta adrenergic receptors that share structural similarity with TAARs. Metastable ligand-binding sites at an extracellular vestibule cause a delay of the adrenergic ligand traversing towards and finally reaching the internal binding site^13,35,36^. A corresponding vestibule has also been described for the interaction of the vasopressin receptor with its ligand^32^.

Very few GPCRs have been examined in this detail so far and, thus, regulation of ligand entry by transient lingering in an external niche or vestibule could conceivably have been overlooked in many cases. Since this mechanism has now been shown in three receptors belonging to two different subfamilies of class A GPCRs (alpha and beta subfamily) we expect that it may well be a much more wide-spread mechanism. It will be fascinating to elucidate how common this mechanism might be among class A GPCRs in general and in their largest clade, i.e. odorant receptors in particular.

## Materials and Methods

### Homology modeling, ligand access path prediction and docking in TAAR13c

TAAR13c homology models were generated using GPCR-I-TASSER. The crystal structures of six homologous receptors were used as templates: two ß2 and two ß1-adrenergic receptors (3sn6R and 2rh1A: 4amjA and 3zpqA, respectively), an adenosine receptor (4eiyQ) and a serotonin receptor (4iarA). The templates were selected automatically by GPCR-I-TASSER. The mutant structures were generated by Chimera^37^ using side chain substitution in the wildtype sequence. We observed no detectable RMSD difference (<0.005) between models generated by these two methods. The receptor model was prepared for docking simulations by adding protons. Charges were assigned to ionizable side chains using the DockPrep function in Chimera^37^. The model was then subjected to energy minimization using ModRefiner^38^. The tunnels in TAAR13c were predicted using MOLE2.0^22^. Probe radius was set to 3.41 Å and the interior threshold to 1.2 keeping the path parameters default. Docking of cadaverine was performed using TomoDock^25^. The pocket was defined by the gating-site residue leading to the internal binding-and-activation site, thus defining a pocket with a depth of 16 Å with D279^6.58^ at the top and D112^3.32^ and D202^5.42^ at the bottom. In order to examine the path of cadaverine while moving towards the binding-and-activation site, we defined a 18 × 18 × 18 Å cubic search space moving from above residue D279^6.58^ towards the internal binding-and-activation site in steps of 1 Å. The ligand was prepared with rotatable and flexible bonds using AutoDock Tools^39^. The resulting conformations were analyzed using PyMol^40^ for visualization and for preparing the figures.

### Heterologous expression of TAAR13c receptor mutants

Cell lines that constitutively expressed either the wildtype TAAR13c receptor or a mutant were generated using a previously established protocol^41^. We used a cell line that had been stably transfected with a gene encoding a variant of the A2-subunit of the olfactory cyclic nucleotide-gated (CNG) ion channel^41,42^. Approximately 10 µg of the respective TAAR13c expression vectors were introduced into 4 × 10^5^ cells by a modified calcium-phosphate method^43^. Stably transfected cells were selected in the presence of the antibiotic G418 (0.8 mg/ml). Expression of TAAR13c was monitored by Western blotting with specific anti-TAAR13c antibodies^17^ and anti-Rhodopsin antibodies (Sigma Aldrich, Taufkirchen, Germany).

### Monitoring functional TAAR13c receptor activity

Activation of TAAR13c by cadaverine and putrescine evokes a rise in intracellular cAMP concentration that activates the CNG channel^41^. This subsequently causes an influx of Ca^2+^ ions through the open channel. Changes in [Ca^2+^]_i_ were monitored with the Ca^2+^-sensitive fluorescent dye Fluo-4. Cells were grown in 96-well plates to a density of approximately 2 × 10^4^ cells per well. Cells were loaded at room temperature with Fluo-4 AM as described previously^44^. After 90 min, the loading solution was substituted for dye-free ECS (extracellular solution; 120 mM NaCl, 5 mM KCl, 2 mM MgCl_2_, 2 mM CaCl_2_, 10 mM HEPES, and 10 mM glucose, pH 7.4 [NaOH]) containing 100 µM IBMX. The plate was transferred into a fluorescence reader (FLUOstar Omega, BMG Labtech, Offenburg, Germany) to monitor Fluo-4 fluorescence. The excitation wavelength was 485 nm. Fluorescence emission was detected at 520 nm. A concentration series of cadaverine or putrescine (0.1 µM to 1 mM) as well as 10 µM NKH477 (adenylyl cyclase activator; positive control) was added once Fluo-4 fluorescence had reached a stable value in each well. The fluorescence signal obtained with NKH477 was set to 100% as an internal standard. Concentration–response curves for mutant TAARs were always performed in parallel with wildtype TAAR13c, and affinity estimates were established from at least three independent experiments with quadruplicate measurements in each experiment.

Data were analyzed and displayed using Prism 5.04 software (GraphPad, San Diego, CA, USA).

### Introduction of mutations into TAAR13c

Wildtype full length TAAR13c with an N-terminal extension of the first 20 amino acids of bovine rhodopsin cloned in pcDNA3.1(−) expression vector^17^ was used for mutagenesis. Point mutations were introduced using the QuikChange^®^ Site-directed mutagenesis kit (Agilent Technologies, Santa Clara, CA, USA). In brief, the PCR reaction was performed using *PfuUltra* High-Fidelity DNA Polymerase with the above described plasmid as a template, along with mutagenic primers and reaction mix. Parental strands, which are methylated in contrast to the PCR products, were selectively digested with *Dpn*1 enzyme and the resulting product was transformed into XL-1 blue supercompetent *E*. *coli* cells by electroporation. Screening of colonies was done by colony PCR using wild type TAAR13c primers. Colonies positive for the desired mutation were grown under standard conditions in LB broth and the plasmids were isolated using a plasmid DNA purification kit from Zymo research (California, USA). All mutations were verified by DNA sequencing. Primers used for mutagenesis were: TAAR13cwt 5′: atggatttatcatcacaagaa 3′: tcaaaccgtaaataaattgat;

D279R 5′: actctctggtgcgtccctacattaac 3′: gttaatgtagggacgcaccagagagt;

D112E 5′: ccggttttgaactgtttctcac 3′: gtgagaaacagttcaaaaccgg;

D279N 5′: actctctggtgaatccctacattaac 3′: gttaatgtagggattcaccagagagt.

## Electronic supplementary material

Supplementary Data
