# Supplementary material for: Full rescue of an inactive olfactory receptor mutant by elimination of an allosteric ligand-gating site
Source: Sci Rep. 2018 Jun 25;8:9631. doi: 10.1038/s41598-018-27790-7 (PMC6018111; doi:10.1038/s41598-018-27790-7)
Supplement: Supplementary file 1 — Supplementary Data [file 41598_2018_27790_MOESM1_ESM.pdf]

Full rescue of an inactive olfactory receptor mutant by elimination of an allosteric ligand-gating site

Kanika Sharma[1], Sabine Balfanz[2]*, Arnd Baumann[2], Sigrun Korsching[1]

[1] Institute of Genetics, Biocenter, University at Cologne, Zülpicherstrasse 47a, 50674 Cologne, Germany

[2] Institute of Complex Systems (ICS-4), Research Center Jülich, 52428 Jülich, Germany

key words: cadaverine, gating, GPCR, site-directed mutagenesis, modeling, docking, calcium imaging

Table of contents:

Supplementary Table 1 EC50 for cadaverine and putrescine for mutant and wildtype TAAR13c ………………………………………………………….. 3

Supplementary Table 1 Interacting residues within the external niche and internal binding-and-activation site ……………………………………………… 3

Supplementary Figure 1 Cavity of the external niche in mutant D279R is narrower than in wildtype TAAR13c due to the bulky side chain of arginine ….. 4

Supplementary Figure 2 Extracellular molecular surfaces of TAAR13c showing potential entry channels ………………………………………………….. 5

Supplementary Table 1 EC50 for cadaverine and putrescine for mutant and wildtype TAAR13c

Wildtype/Mutant	EC50 (μM, mean+/−SEM)	
	cadaverine	putrescine
TAAR13c wt for D112E/D279N	35.6 ± 8.9	>1000
D112E/D279N	58.8 ± 6.1	>1000
TAAR13c wt for D279R	78.0 ± 12.0	>1000
D279R	15.1 ± 3.9	>100

Supplementary Table 2 Interacting residues within the external niche and internal binding-and-activation site

Amino acid residues predicted by docking to interact with cadaverine. Mutant TAAR13c is indicated by the amino acid exchange, e.g. D279R, D112E/D279N

Wildtype/ Mutant	***Interacting Residues***	
	External niche	***Internal binding site***
TAAR13c	E175, Y193, F194, N195, A196, S199, D279, P280, Y288, F291	D112, L113, T116, D202, T203, F272
D279R	E175, Y193, F194, N195, A196, S199, R279, P280, N283	D112, L113, T116, D202, T203, T206, F272
D112E/D279N	E175, L192, Y193, F194, N195, A196, S199, N279, P280, N283	L113, T116, D202, T203, T206, W269, F273

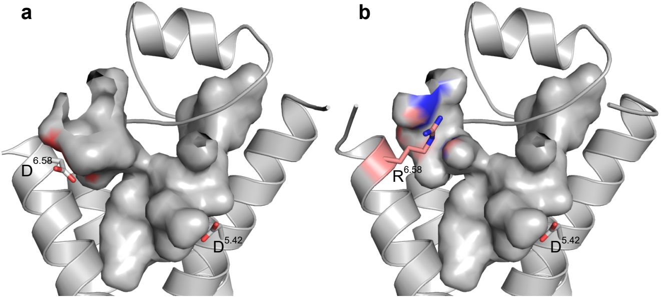

Supplementary Figure 1 Cavity of the external niche in mutant D279R is narrower than in wildtype TAAR13c due to the bulky side chain of arginine.

(a) Gating residue D279 (position 6.58) lining the cavity of the external niche. (b) Gating-site mutated to arginine, D279R (position 6.58) (salmon) with a bulky side chain narrows the cavity and possibly limits cadaverine exploration time in the external niche.

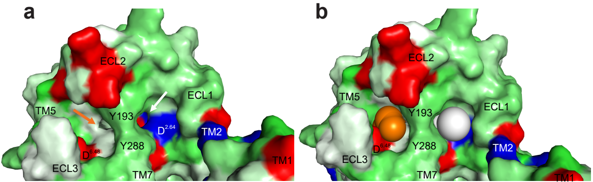

Supplementary Figure 2 Extracellular molecular surfaces of TAAR13c showing potential entry channels

Surface view from the extracellular surface; red, acidic residues; blue, basic residues; green, polar residues; white, hydrophobic residues. (a) Arrows pointing to openings in TAAR13c towards internal binding site. Negatively charged $D^{6.58}$ lining the left route along with electronegative environment of ECL2 is favorable for attracting cadaverine. (b) The accessible tunnels generated by MOLE 2.0 in orange and white.
